# Supplementary material for: Integrative proteomic and lipidomic analysis of GNB1 and SCARB2 knockdown in human subcutaneous adipocytes
Source: PLoS One. 2025 Mar 24;20(3):e0319163. doi: 10.1371/journal.pone.0319163 (PMC11932494; doi:10.1371/journal.pone.0319163)
Supplement: S1 Fig — (DOCX) [file pone.0319163.s001.docx]

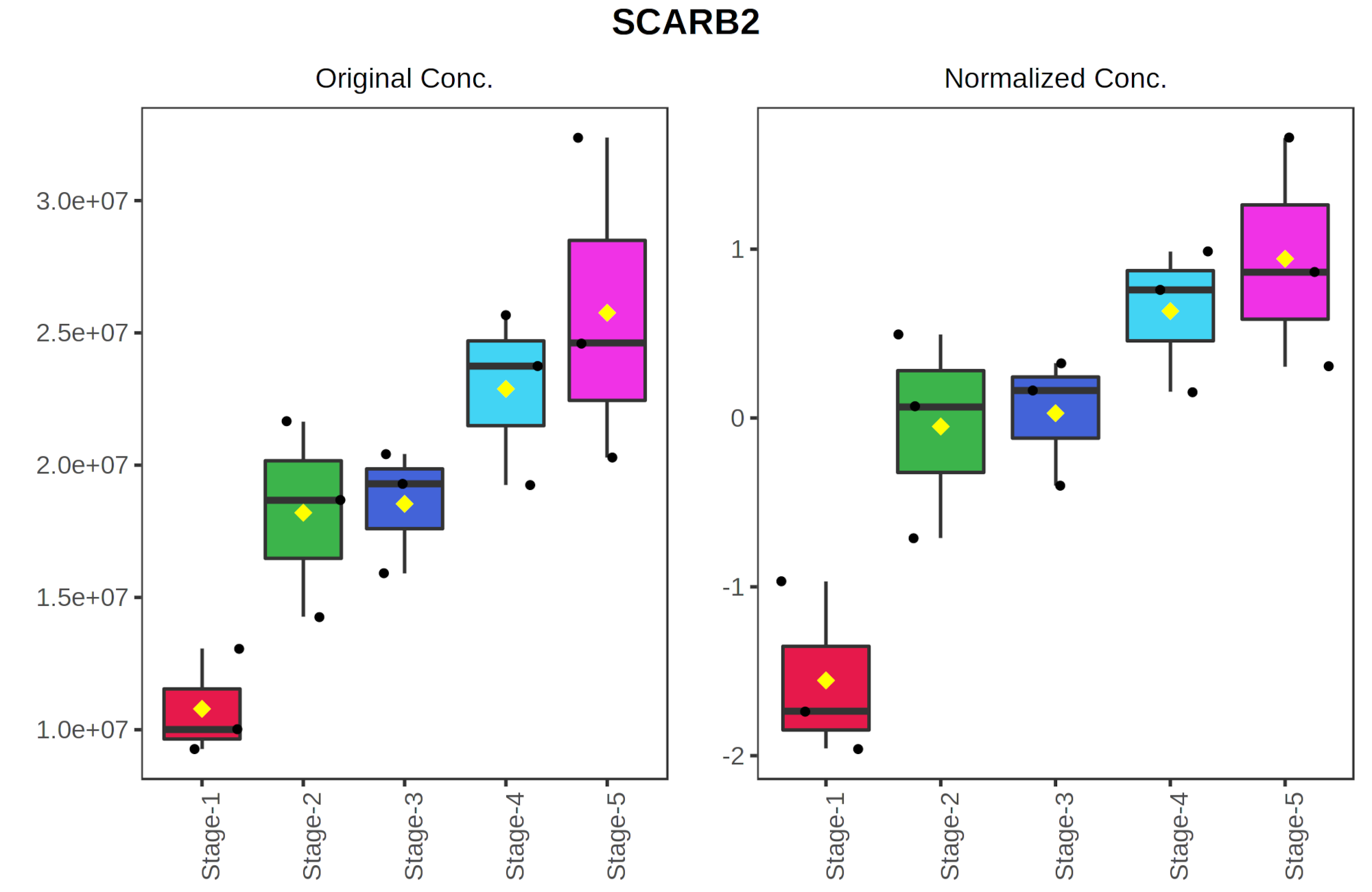

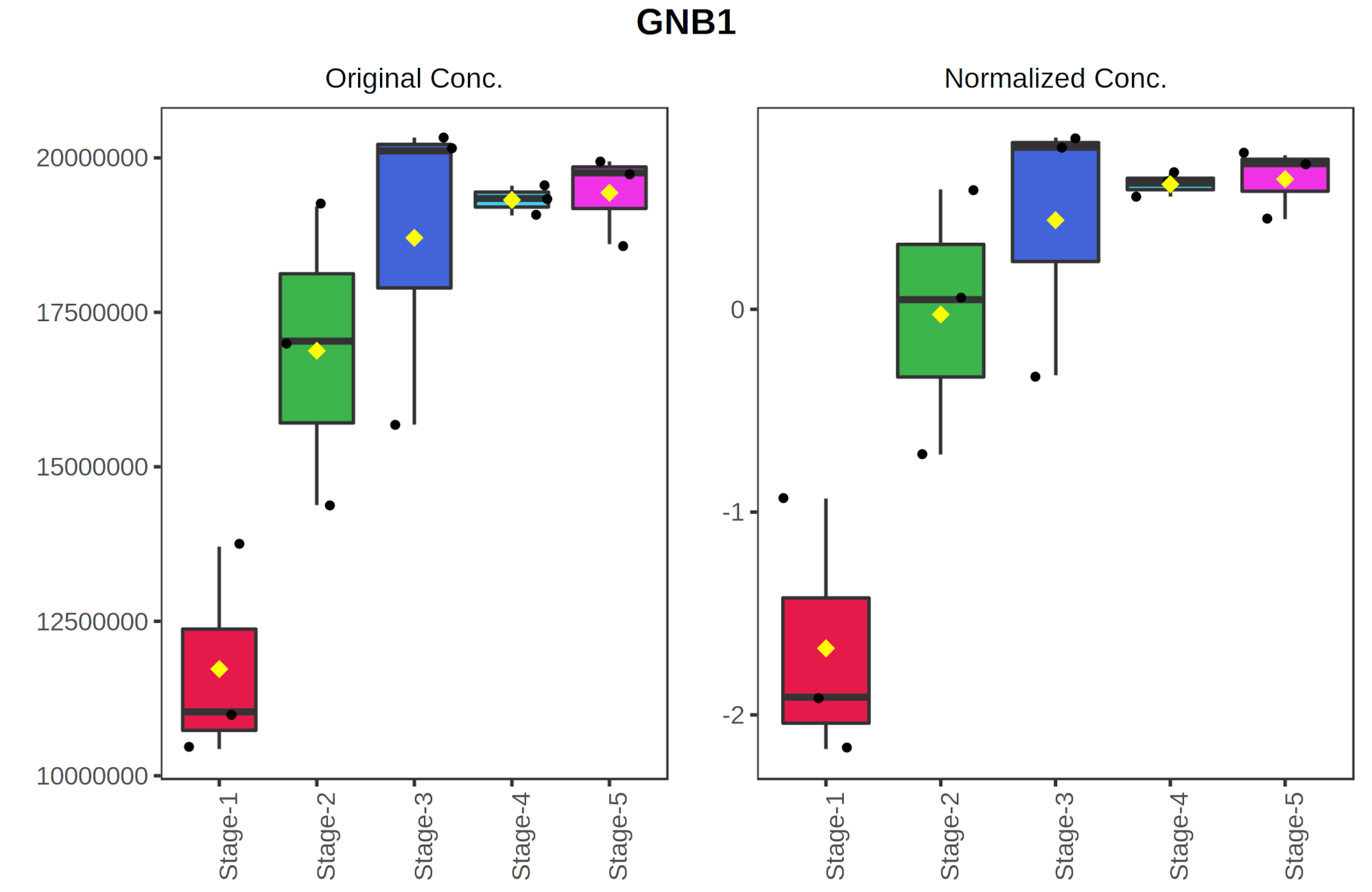


**GNB1**

Normalized concentration

**SCARB2**

Normalized concentration

**S1 Fig. Expression profiles of GNB1 and SCARB2 during subcutaneous adipocyte differentiation and maturation.**

Protein abundances were measured by mass spectrometry and visualized as box-and-whisker plots using MetaboAnalyst 6.0. Stage-1: subcutaneous preadipocytes; stage-2: following induction of differentiation into adipocytes; stage-3 to stage-5: from the initiation of lipid droplet formation to mature subcutaneous adipocytes. Both proteins showed increased expression levels from the pre-differentiation stage through to the maturation stage. Data was derived from our previous proteomic analysis [21].
