# Supplementary material for: Integrative proteomic and lipidomic analysis of GNB1 and SCARB2 knockdown in human subcutaneous adipocytes
Source: PLoS One. 2025 Mar 24;20(3):e0319163. doi: 10.1371/journal.pone.0319163 (PMC11932494; doi:10.1371/journal.pone.0319163)
Supplement: S2 Fig — (DOCX) [file pone.0319163.s002.docx]

**(A)**


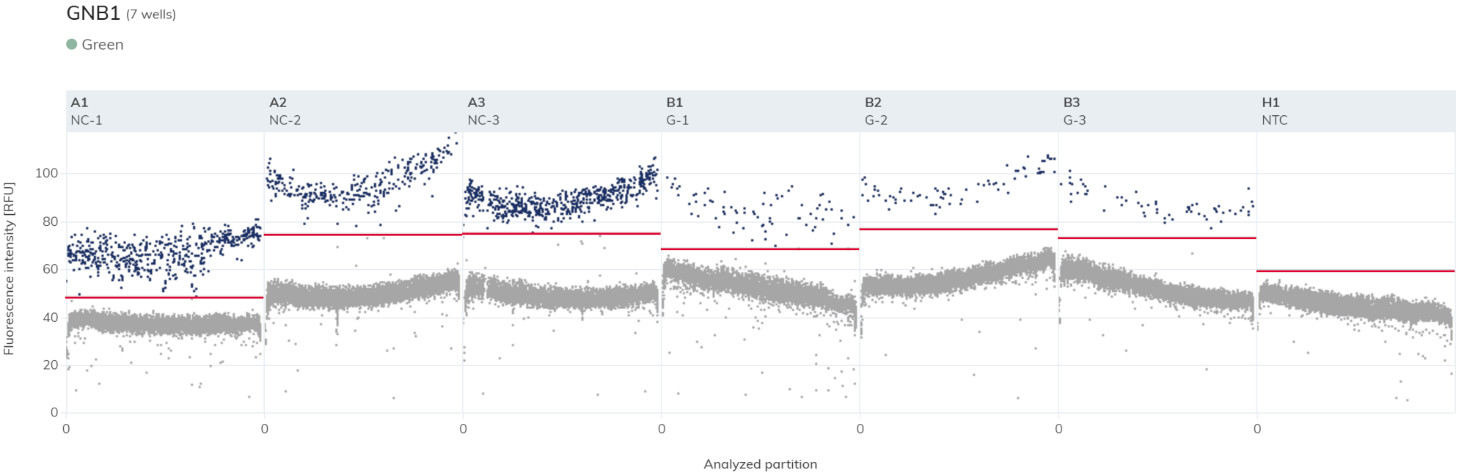


siRNA_negative control siRNA_*GNB1*

NTC

**(B)**


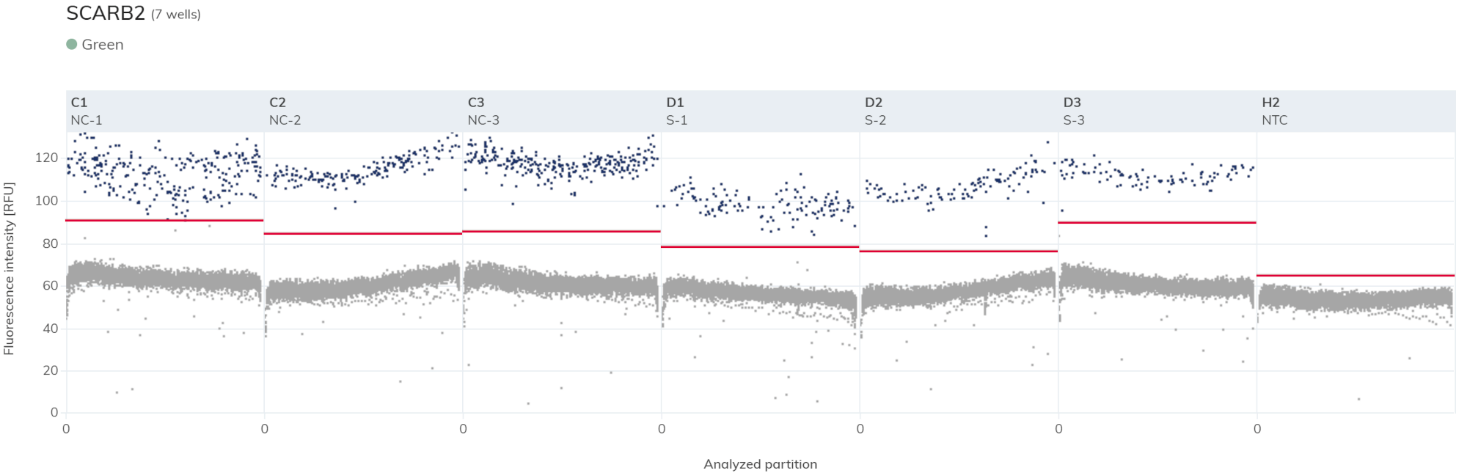


siRNA_negative control siRNA_*SCARB2*

NTC

**(C)**


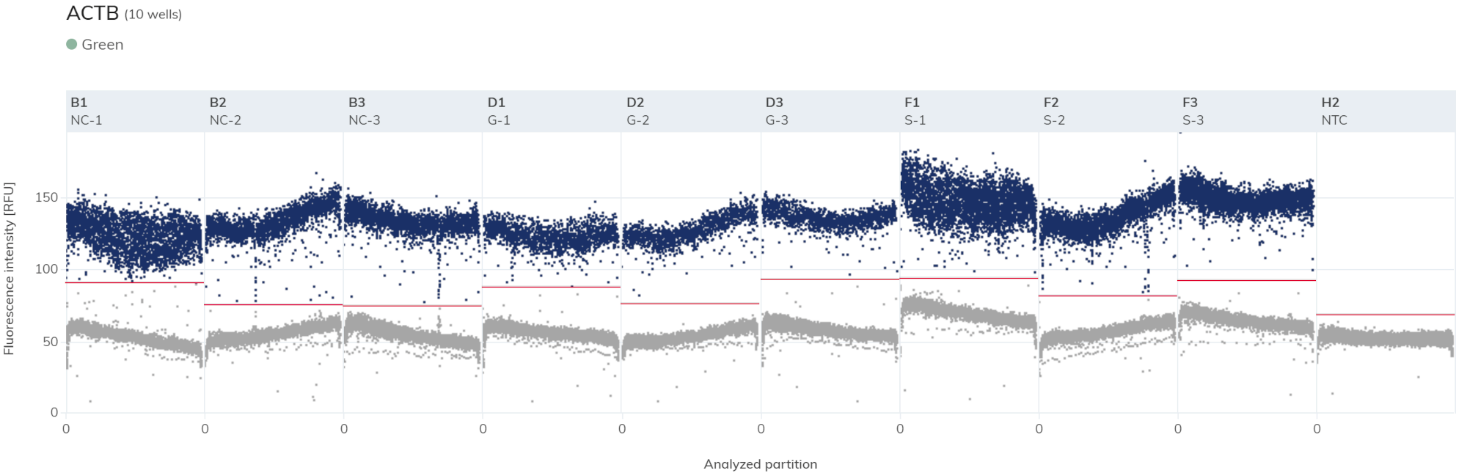


siRNA_negative control

siRNA_*GNB1* siRNA_*SCARB2*

NTC

**S2 Fig. Visualization of target gene amplification with absolute quantification analysis.**

The amplification of the target gene was demonstrated in 1D scatter plots using the absolute quantification analysis using (A) *GNB1*, (B) *SCARB2*, and (C) *ACTB* primer sets. The EvaGreen fluorescent signal was plotted on the x-axis as an analyzed partition and on the y-axis as fluorescence intensity [RFU]. The gray bars represent the no-template control (NTC) for digital PCR. The navy colors indicate positive partitions, while the light gray colors indicate negative partitions. Abbreviations: NC, siRNA-negative control; G, siRNA-G protein subunit beta 1 (*GNB1*); S, siRNA-scavenger receptor class B member 2 (*SCARB2*); NTC, no-template control.
