# Supplementary material for: Integrative proteomic and lipidomic analysis of GNB1 and SCARB2 knockdown in human subcutaneous adipocytes
Source: PLoS One. 2025 Mar 24;20(3):e0319163. doi: 10.1371/journal.pone.0319163 (PMC11932494; doi:10.1371/journal.pone.0319163)
Supplement: S3 Fig — (DOCX) [file pone.0319163.s003.docx]

siRNA-negative control

siRNA-*GNB1*

siRNA-*SCARB2*


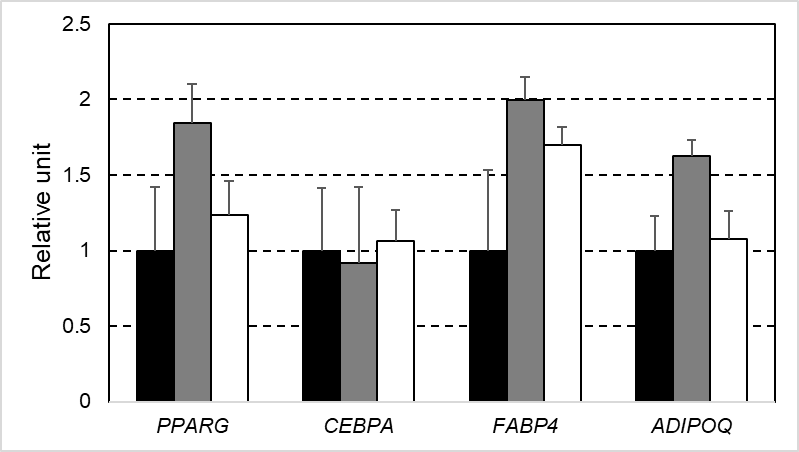


**S3 Fig. Comparison of adipogenic marker expression in *GNB1* and *SCARB2* knockdown cells using quantitative real-time PCR.**

Gene expression was normalized to *ACTB* using the 2^−ΔΔCq^ method. Data are presented as mean (SD) of three independent experiments. No significant differences were observed between groups (FDR adjusted *P* > 0.05). Abbreviations: *PPARG*, peroxisome proliferator activated receptor gamma; *CEBPA*, CCAAT enhancer binding protein alpha; *FABP4*, fatty acid binding protein 4; *ADIPOQ*, adiponectin, C1Q and collagen domain containing; SD, standard deviation.
