# Supplementary material for: Integrative proteomic and lipidomic analysis of GNB1 and SCARB2 knockdown in human subcutaneous adipocytes
Source: PLoS One. 2025 Mar 24;20(3):e0319163. doi: 10.1371/journal.pone.0319163 (PMC11932494; doi:10.1371/journal.pone.0319163)
Supplement: S4 Fig — (PDF) [file pone.0319163.s004.pdf]

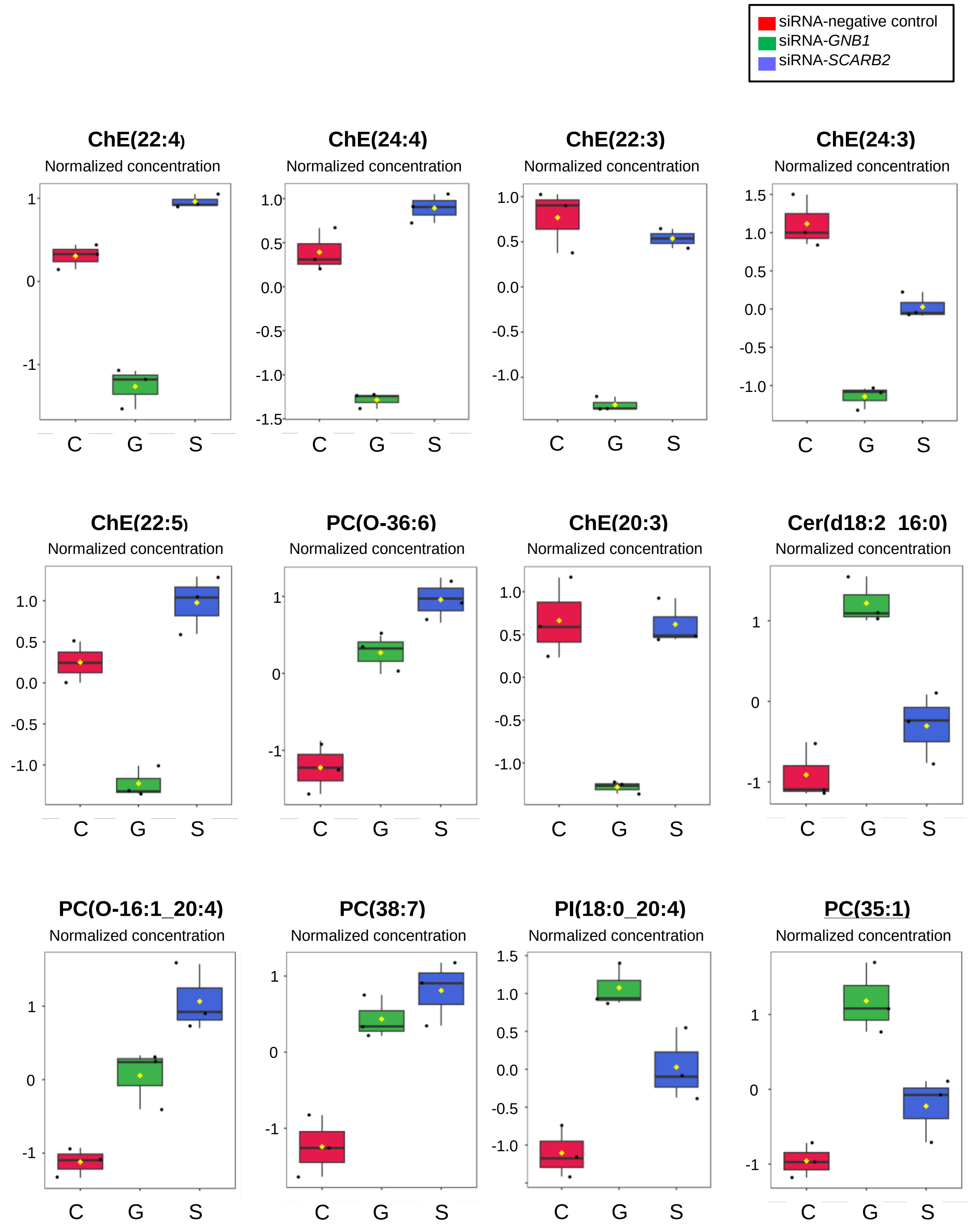

S4 Fig. Analysis of significant lipid species alterations following knockdown of *GNB1* and *SCARB2*. Box and whisker plots of the top 12 lipid species that remained significant after FDR correction (adjusted $P < 0.05$). The red, green, and blue boxes indicate the siRNA-negative control, siRNA-*GNB1*, and siRNA-*SCARB2*, respectively. Abbreviations: C, siRNA-negative control; G, siRNA-G protein subunit beta 1 (*GNB1*); S, siRNA-scavenger receptor class B member 2 (*SCARB2*); FDR, false discovery rate.
