# Supplementary material for: Integrative proteomic and lipidomic analysis of GNB1 and SCARB2 knockdown in human subcutaneous adipocytes
Source: PLoS One. 2025 Mar 24;20(3):e0319163. doi: 10.1371/journal.pone.0319163 (PMC11932494; doi:10.1371/journal.pone.0319163)
Supplement: S5 Fig — (PDF) [file pone.0319163.s005.pdf]

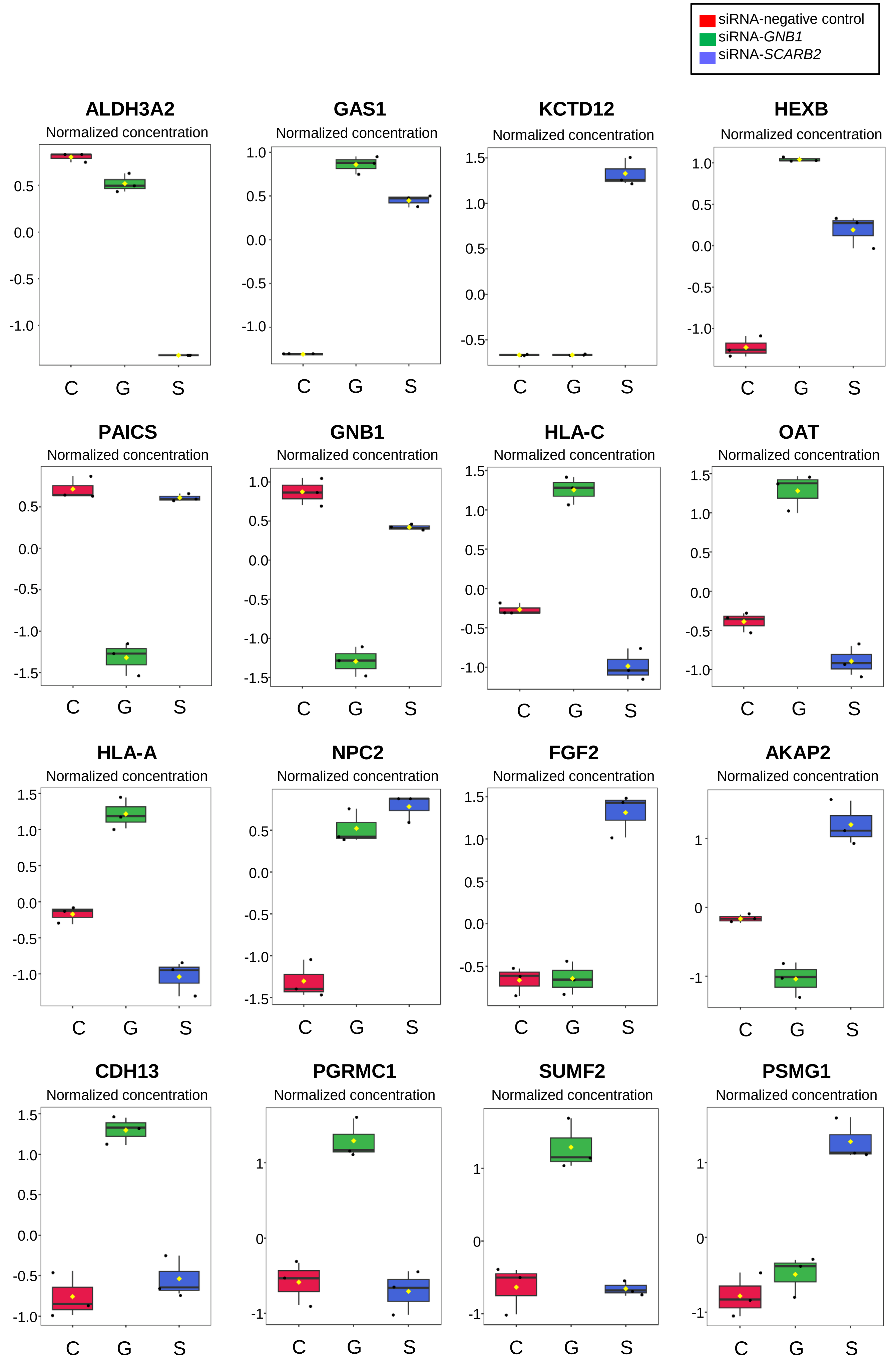

siRNA-negative control
siRNA-GNB1
siRNA-SCARB2
ALDH3A2
GAS1
KCTD12
HEXB
PAICS
GNB1
HLA-C
OAT
HLA-A
NPC2
FGF2
AKAP2
CDH13
PGRMC1
SUMF2
PSMG1
Normalized concentration
C
G
S

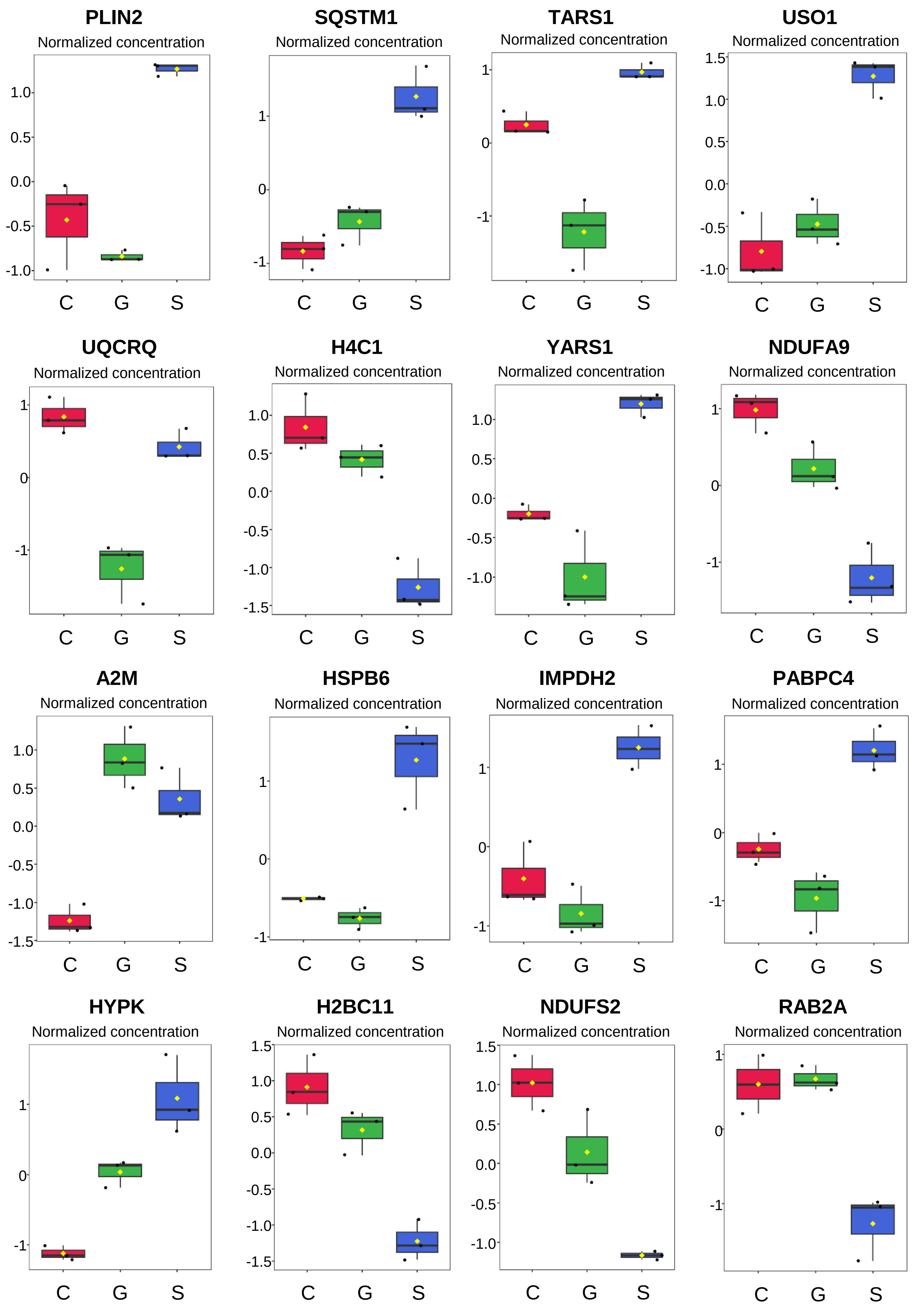
PLIN2
Normalized concentration
1.0
0.5
0.0
-0.5
-1.0
C
G
S
SQSTM1
Normalized concentration
1
0
-1
C
G
S
TARS1
Normalized concentration
1
0
-1
C
G
S
USO1
Normalized concentration
1.5
1.0
0.5
0.0
-0.5
-1.0
C
G
S
UQCRQ
Normalized concentration
1
0
-1
C
G
S
H4C1
Normalized concentration
1.0
0.5
0.0
-0.5
-1.0
-1.5
C
G
S
YARS1
Normalized concentration
1.0
0.5
0.0
-0.5
-1.0
C
G
S
NDUFA9
Normalized concentration
1
0
-1
C
G
S
A2M
Normalized concentration
1.0
0.5
0.0
-0.5
-1.0
-1.5
C
G
S
HSPB6
Normalized concentration
1
0
-1
C
G
S
IMPDH2
Normalized concentration
1
0
-1
C
G
S
PABPC4
Normalized concentration
1
0
-1
C
G
S
HYPK
Normalized concentration
1
0
-1
C
G
S
H2BC11
Normalized concentration
1.5
1.0
0.5
0.0
-0.5
-1.0
-1.5
C
G
S
NDUFS2
Normalized concentration
1.5
1.0
0.5
0.0
-0.5
-1.0
C
G
S
RAB2A
Normalized concentration
1
0
-1
C
G
S

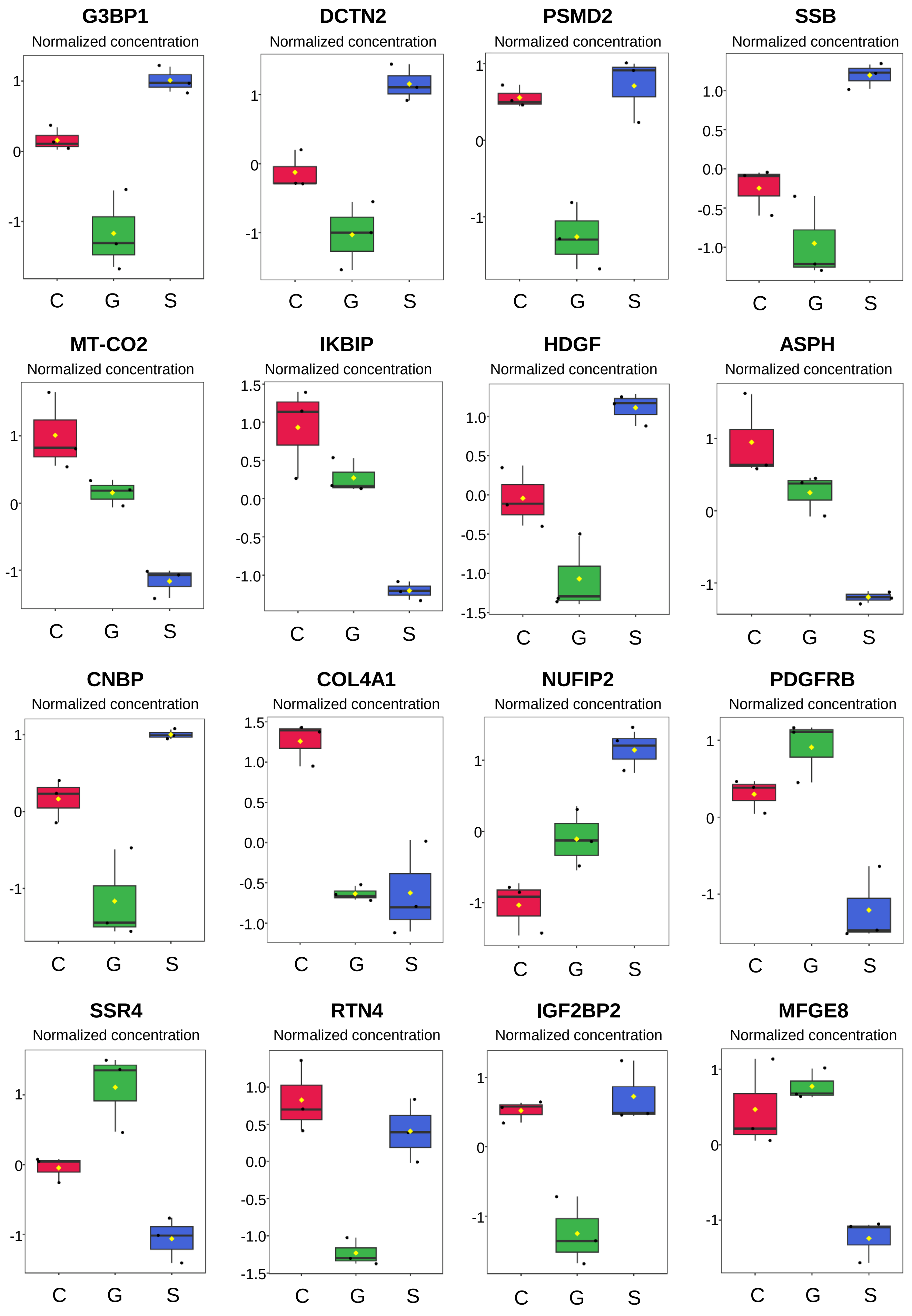
G3BP1
Normalized concentration
1
0
-1
C
G
S
DCTN2
Normalized concentration
1
0
-1
C
G
S
PSMD2
Normalized concentration
1
0
-1
C
G
S
SSB
Normalized concentration
1.0
0.5
0.0
-0.5
-1.0
C
G
S
MT-CO2
Normalized concentration
1
0
-1
C
G
S
IKBIP
Normalized concentration
1.5
1.0
0.5
0.0
-0.5
-1.0
C
G
S
HDGF
Normalized concentration
1.0
0.5
0.0
-0.5
-1.0
-1.5
C
G
S
ASPH
Normalized concentration
1
0
-1
C
G
S
CNBP
Normalized concentration
1
0
-1
C
G
S
COL4A1
Normalized concentration
1.5
1.0
0.5
0.0
-0.5
-1.0
C
G
S
NUFIP2
Normalized concentration
1
0
-1
C
G
S
PDGFRB
Normalized concentration
1
0
-1
C
G
S
SSR4
Normalized concentration
1
0
-1
C
G
S
RTN4
Normalized concentration
1.0
0.5
0.0
-0.5
-1.0
-1.5
C
G
S
IGF2BP2
Normalized concentration
1
0
-1
C
G
S
MFGE8
Normalized concentration
1
0
-1
C
G
S

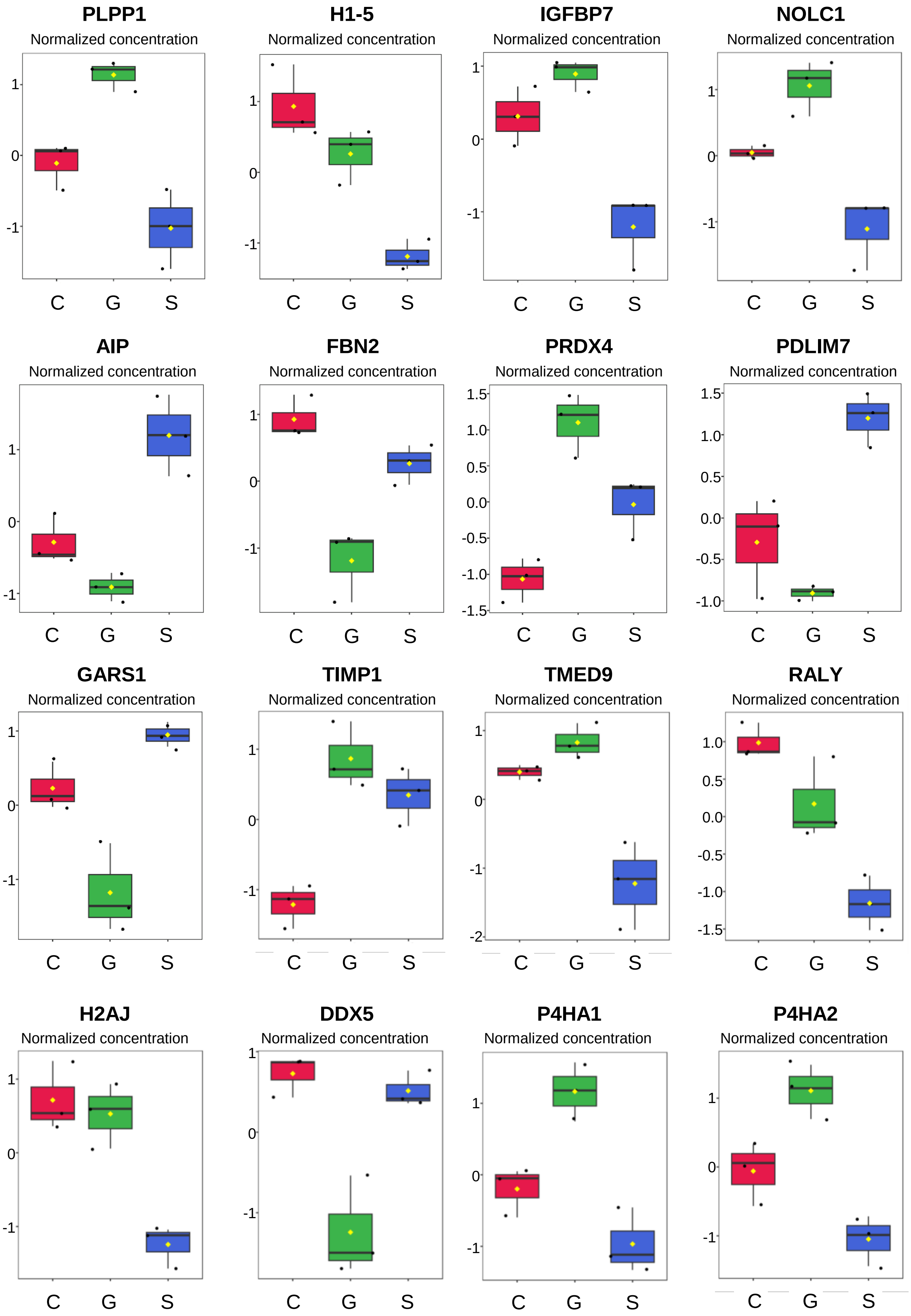
PLPP1
Normalized concentration
1
0
-1
C G S
H1-5
Normalized concentration
1
0
-1
C G S
IGFBP7
Normalized concentration
1
0
-1
C G S
NOLC1
Normalized concentration
1
0
-1
C G S
AIP
Normalized concentration
1
0
-1
C G S
FBN2
Normalized concentration
1
0
-1
C G S
PRDX4
Normalized concentration
1.5
1.0
0.5
0.0
-0.5
-1.0
-1.5
C G S
PDLIM7
Normalized concentration
1.5
1.0
0.5
0.0
-0.5
-1.0
C G S
GARS1
Normalized concentration
1
0
-1
C G S
TIMP1
Normalized concentration
1
0
-1
C G S
TMED9
Normalized concentration
1
0
-1
-2
C G S
RALY
Normalized concentration
1.0
0.5
0.0
-0.5
-1.0
-1.5
C G S
H2AJ
Normalized concentration
1
0
-1
C G S
DDX5
Normalized concentration
1
0
-1
C G S
P4HA1
Normalized concentration
1
0
-1
C G S
P4HA2
Normalized concentration
1
0
-1
C G S

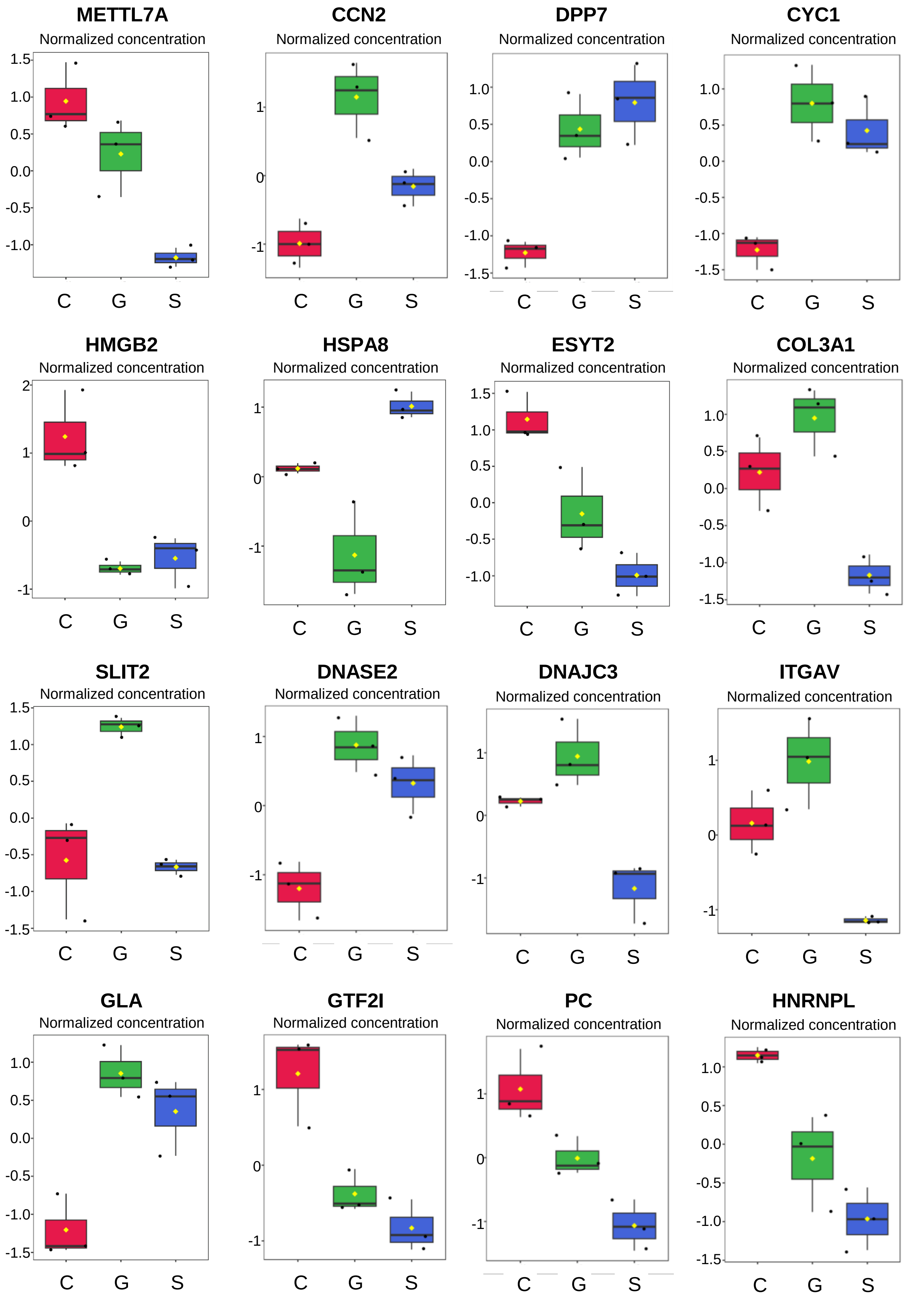
METTL7A
Normalized concentration
1.5
1.0
0.5
0.0
-0.5
-1.0
C
G
S
CCN2
Normalized concentration
1
0
-1
C
G
S
DPP7
Normalized concentration
1.0
0.5
0.0
-0.5
-1.0
-1.5
C
G
S
CYC1
Normalized concentration
1.0
0.5
0.0
-0.5
-1.0
-1.5
C
G
S
HMGB2
Normalized concentration
2
1
0
-1
C
G
S
HSPA8
Normalized concentration
1
0
-1
C
G
S
ESYT2
Normalized concentration
1.5
1.0
0.5
0.0
-0.5
-1.0
C
G
S
COL3A1
Normalized concentration
1.0
0.5
0.0
-0.5
-1.0
-1.5
C
G
S
SLIT2
Normalized concentration
1.5
1.0
0.5
0.0
-0.5
-1.0
-1.5
C
G
S
DNASE2
Normalized concentration
1
0
-1
C
G
S
DNAJC3
Normalized concentration
1
0
-1
C
G
S
ITGAV
Normalized concentration
1
0
-1
C
G
S
GLA
Normalized concentration
1.0
0.5
0.0
-0.5
-1.0
-1.5
C
G
S
GTF2I
Normalized concentration
1
0
-1
C
G
S
PC
Normalized concentration
1
0
-1
C
G
S
HNRNPL
Normalized concentration
1.0
0.5
0.0
-0.5
-1.0
-1.5
C
G
S

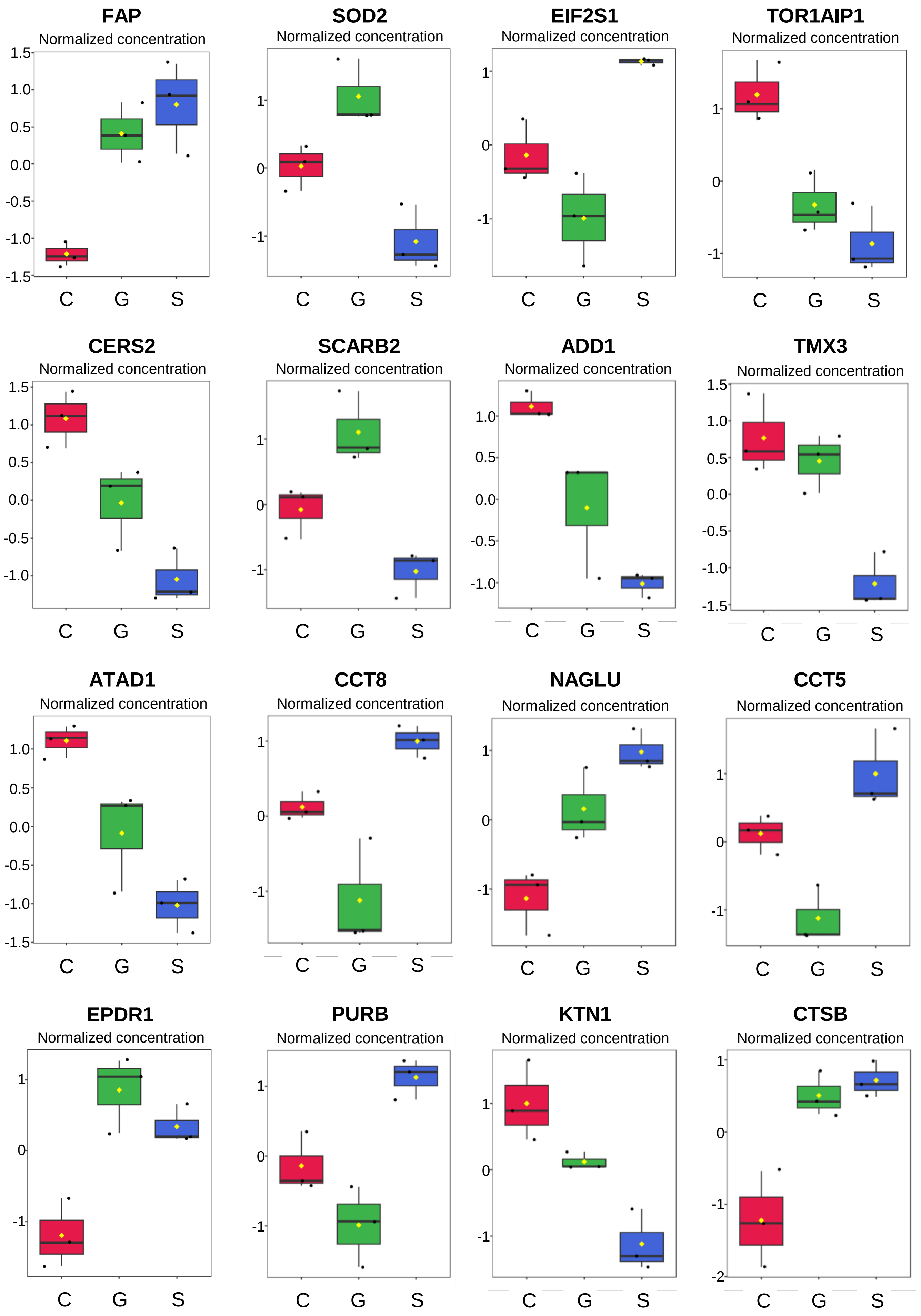
FAP
Normalized concentration
1.5
1.0
0.5
0.0
-0.5
-1.0
-1.5
C G S
SOD2
Normalized concentration
1
0
-1
C G S
EIF2S1
Normalized concentration
1
0
-1
C G S
TOR1AIP1
Normalized concentration
1
0
-1
C G S
CERS2
Normalized concentration
1.5
1.0
0.5
0.0
-0.5
-1.0
C G S
SCARB2
Normalized concentration
1
0
-1
C G S
ADD1
Normalized concentration
1.0
0.5
0.0
-0.5
-1.0
C G S
TMX3
Normalized concentration
1.5
1.0
0.5
0.0
-0.5
-1.0
-1.5
C G S
ATAD1
Normalized concentration
1.0
0.5
0.0
-0.5
-1.0
-1.5
C G S
CCT8
Normalized concentration
1
0
-1
C G S
NAGLU
Normalized concentration
1
0
-1
C G S
CCT5
Normalized concentration
1
0
-1
C G S
EPDR1
Normalized concentration
1
0
-1
C G S
PURB
Normalized concentration
1
0
-1
C G S
KTN1
Normalized concentration
1
0
-1
C G S
CTSB
Normalized concentration
1
0
-1
-2
C G S

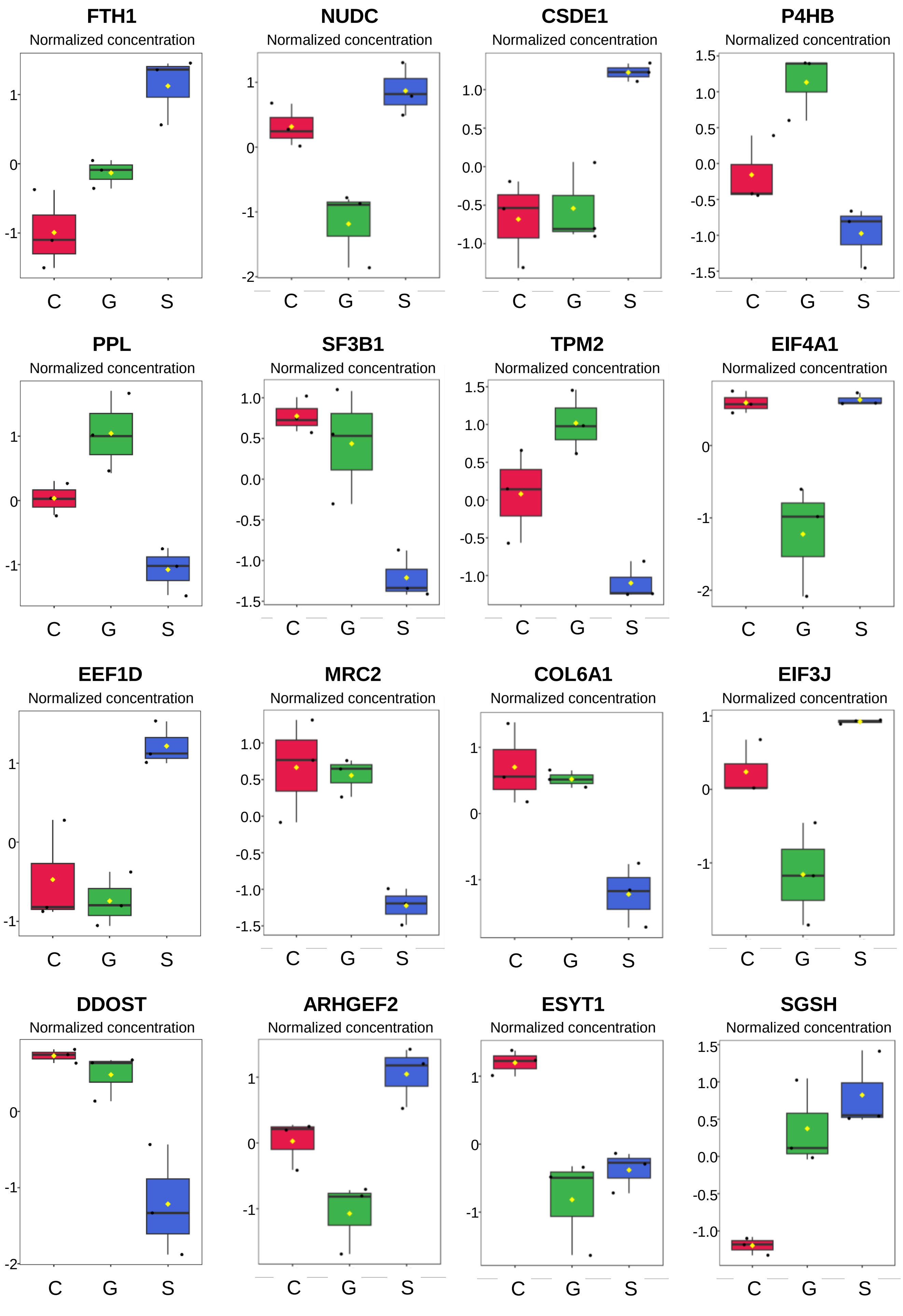

FTH1
NUDC
CSDE1
P4HB
PPL
SF3B1
TPM2
EIF4A1
EEF1D
MRC2
COL6A1
EIF3J
DDOST
ARHGEF2
ESYT1
SGSH
Normalized concentration
C
G
S

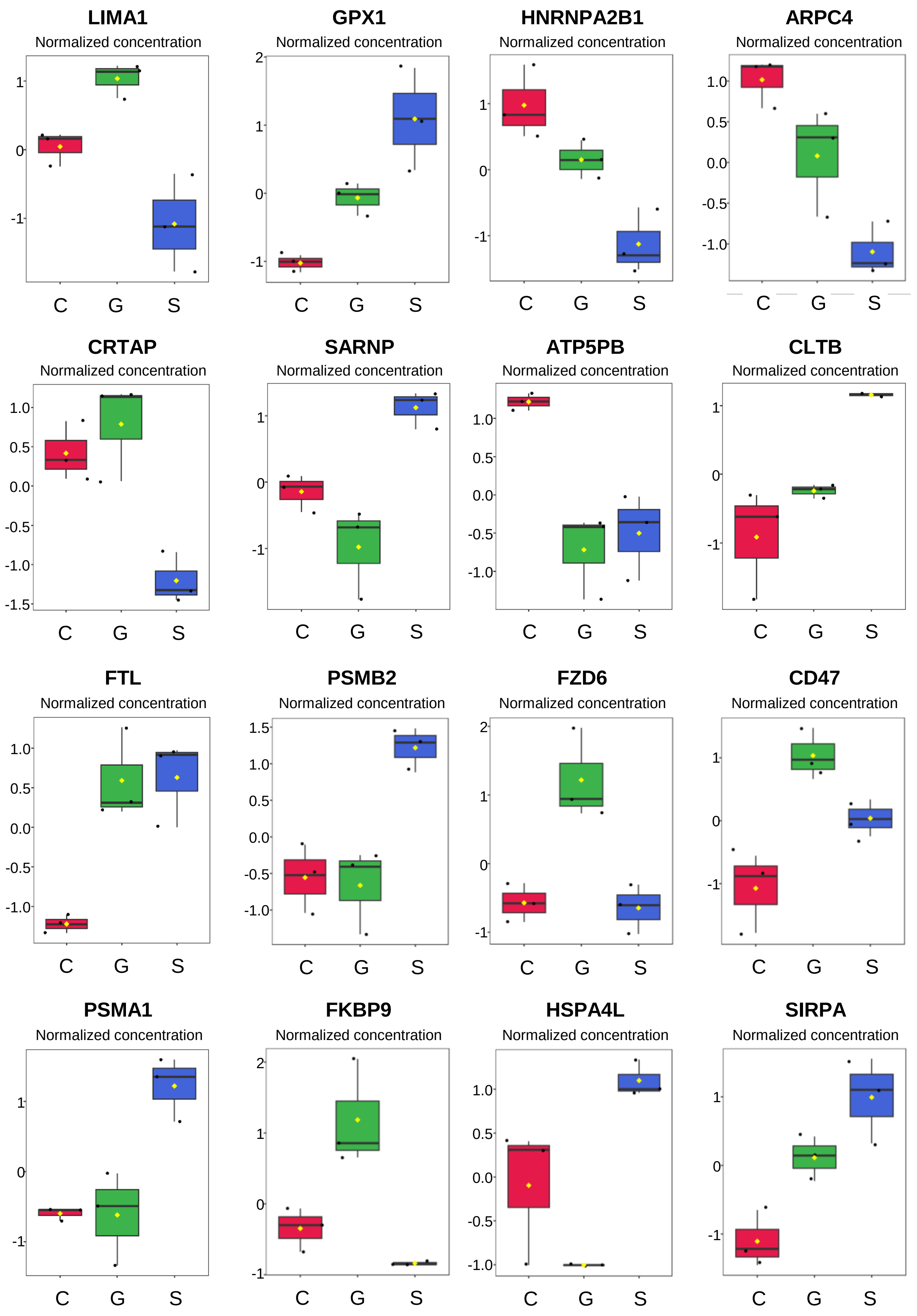
LIMA1
Normalized concentration
1
0
-1
C G S
GPX1
Normalized concentration
2
1
0
-1
C G S
HNRNPA2B1
Normalized concentration
1
0
-1
C G S
ARPC4
Normalized concentration
1.0
0.5
0.0
-0.5
-1.0
C G S
CRTAP
Normalized concentration
1.0
0.5
0.0
-0.5
-1.0
-1.5
C G S
SARNP
Normalized concentration
1
0
-1
C G S
ATP5PB
Normalized concentration
1.0
0.5
0.0
-0.5
-1.0
C G S
CLTB
Normalized concentration
1
0
-1
C G S
FTL
Normalized concentration
1.0
0.5
0.0
-0.5
-1.0
C G S
PSMB2
Normalized concentration
1.5
1.0
0.5
0.0
-0.5
-1.0
C G S
FZD6
Normalized concentration
2
1
0
-1
C G S
CD47
Normalized concentration
1
0
-1
C G S
PSMA1
Normalized concentration
1
0
-1
C G S
FKBP9
Normalized concentration
2
1
0
-1
C G S
HSPA4L
Normalized concentration
1.0
0.5
0.0
-0.5
-1.0
C G S
SIRPA
Normalized concentration
1
0
-1
C G S

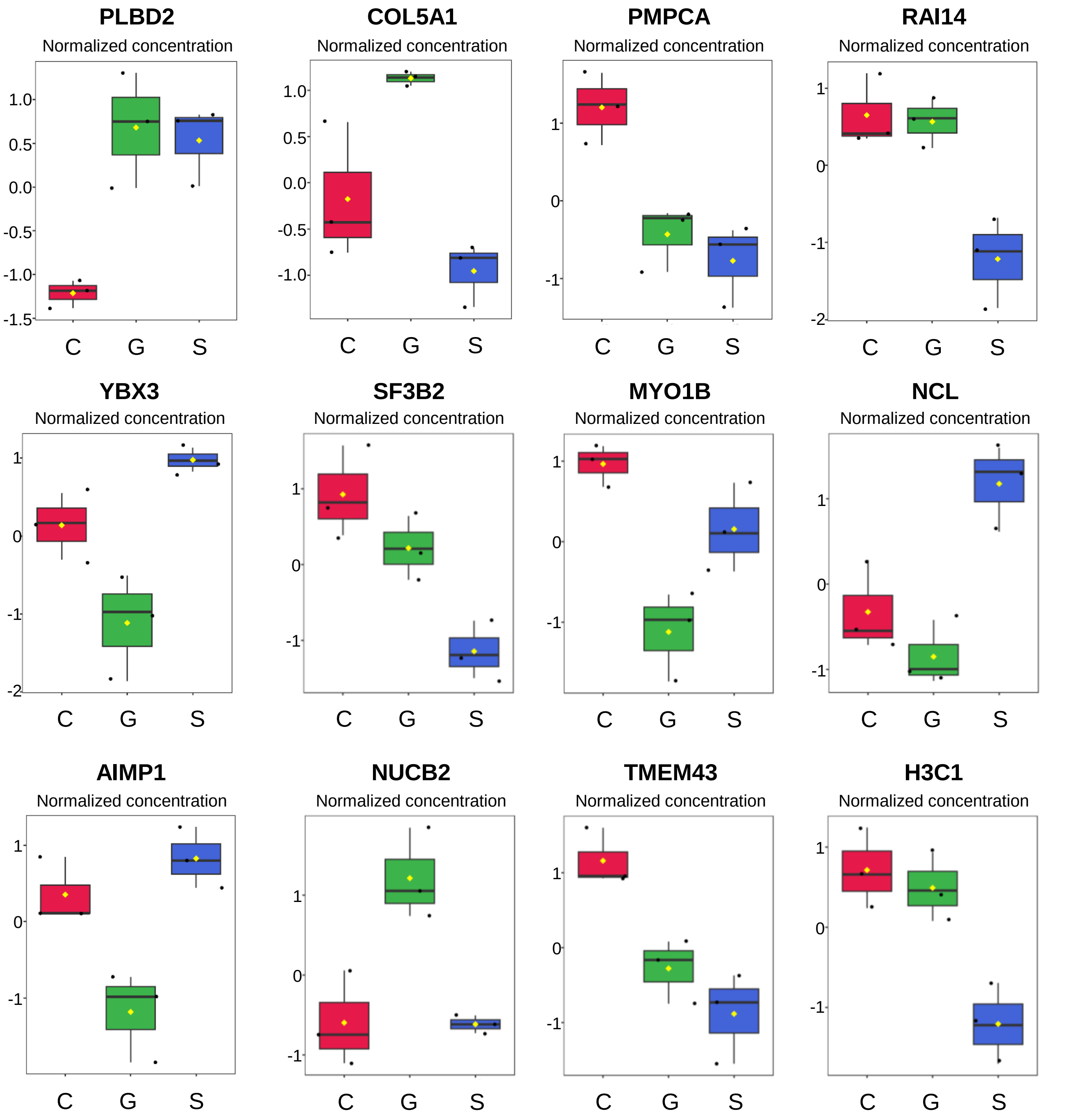

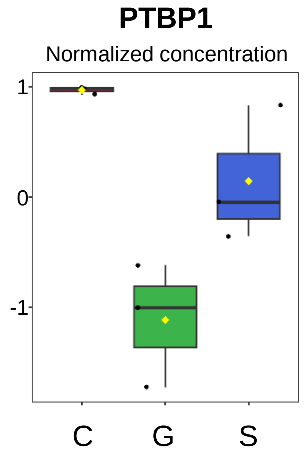

S5 Fig. Analysis of significant protein alterations following knockdown of *GNB1* and *SCARB2*.

Box and whisker plots of the top 141 proteins that remained significant after FDR correction (adjusted $P < 0.05$). The red, green, and blue boxes indicate the siRNA-negative control, siRNA-*GNB1*, and siRNA-*SCARB2*, respectively. Abbreviations: C, siRNA-negative control; G, siRNA-G protein subunit beta 1 (*GNB1*); S, siRNA- scavenger receptor class B member 2 (*SCARB2*); FDR, false discovery rate.
