# Supplementary material for: Integrative proteomic and lipidomic analysis of GNB1 and SCARB2 knockdown in human subcutaneous adipocytes
Source: PLoS One. 2025 Mar 24;20(3):e0319163. doi: 10.1371/journal.pone.0319163 (PMC11932494; doi:10.1371/journal.pone.0319163)
Supplement: S1 Table — (DOCX) [file pone.0319163.s006.docx]

**S1 Table.** **Primer sequences used for digital PCR and quantitative real-time PCR, and the estimated amplicon sizes.**

| **Gene** | **GenBank**  **Accession**  **(variant No.)** | **Gene ID** | **Forward and reverse primer** | **Forward and reverse**  **primer sequence**  **(5′ → 3′)** | **Estimated amplicon size (bp)** | **Location human genome sequence (NCBI GRCh37)** |
| --- | --- | --- | --- | --- | --- | --- |
| *GNB1* | NM_002074.5  (1) | 2782 | G_F2  G_R2 | CAACGTCTGGGATGCACTCAA  CTTGAGGAAGCTATCCCAGGA | 130 | 1,720,506-1,720,526  1,718,782-1,718,802 |
| *SCARB2* | NM_005506.4  (1) | 950 | S_F1  S_R1 | GTCTTCCAGAAGGCTGTAGAC  GATCTCCTCTGGATTGGTGAC | 144 | 77,134,595-77,134,615  77,116,910-77,116,930 |
| *PPARG* | NM_015869.5  (2) | 5468 | P_F2  P_R2 | GGAGTTCATGCTTGTGAAGGAT  CTCCGCCAACAGCTTCTCCTT | 219 | 12,422,964-12,422,985  12,447,413-12,447,433 |
| *CEBPA* | NM_004364.5  (1) | 1050 | C_F2  C_R2 | GGACAAGAACAGCAACGAGTAC  GTCATTGTCACTGGTCAGCTC | 133 | 33,792,466-33,792,487  33,792,355-33,792,375 |
| *FABP4* | NM_001442.3 | 2167 | F_F2  F_R2 | CACCATAACCTTAGATGGGGGT  CATAAACTCTCGTGGAAGTGAC | 140 | 82,391,727-82,391,748  82,391,111-82,391,132 |
| *ADIPOQ* | NM_001177800.2  (1) | 9370 | A_F2  A_R2 | CAGGCCGTGATGGCAGAGAT  TTTCACCGATGTCTCCCTTAGG | 130 | 186,571,005-186,571,024  186,571,984-186,572,005 |
| *ACTB* | NM_001101.5 | 60 | B_F2  B_R2 | ACCTTCAACTCCATCATGAAGT  CAGGAGGAGCAATGATCTTGAT | 169 | 5,567,766-5,567,787  5,567,507-5,567,640 |
| *GAPDH* | NM_002046.7  (1) | 2597 | D_F1  D_R1 | GAGTCAACGGATTTGGTCGT  GGGTGGAATCATATTGGAACAT | 138 | 6,644,018-6,645,669  6,645,856-6,645-877 |

Abbreviations: *GNB1,* G protein subunit beta 1; *SCARB2,* scavenger receptor class B member 2; *PPARG*, peroxisome proliferator activated receptor gamma; *CEBPA*, CCAAT enhancer binding protein alpha; *FABP4*, fatty acid binding protein 4; *ADIPOQ*, adiponectin, C1Q and collagen domain containing; *ACTB,* actin beta; *GAPDH*, glyceraldehyde-3-phosphate dehydrogenase.
