# Supplementary material for: Integrative proteomic and lipidomic analysis of GNB1 and SCARB2 knockdown in human subcutaneous adipocytes
Source: PLoS One. 2025 Mar 24;20(3):e0319163. doi: 10.1371/journal.pone.0319163 (PMC11932494; doi:10.1371/journal.pone.0319163)
Supplement: S2 Table — (DOCX) [file pone.0319163.s007.docx]

**S2 Table. Digital PCR analysis program.**

| dPCR platform | QIAcuity One 5-Plex System (Qiagen) |
| --- | --- |
| Plate Type | QIAcuity Nanoplate 8.5K 24-well (Cat No. 250011, Lot No. 5694100115) |
| Wells | 24 |
| Input reaction volume (µL) | 12 |
| Number of partitions | Approximately 8,500 |
| Partition volume (nL) | 0.34 |
| Master mix | QIAcuity EG PCR Kit (Cat No. 250112, Lot No. 166051688) |
| Analysis software | QIAcuity Software Suite version 2.2.0.26 |
