# Supplementary material for: Integrative proteomic and lipidomic analysis of GNB1 and SCARB2 knockdown in human subcutaneous adipocytes
Source: PLoS One. 2025 Mar 24;20(3):e0319163. doi: 10.1371/journal.pone.0319163 (PMC11932494; doi:10.1371/journal.pone.0319163)
Supplement: S3 Table — (DOCX) [file pone.0319163.s008.docx]

**S3 Table. dMIQE checklist for digital PCR experiments.**

| **ITEM TO CHECK** | **PROVIDED** | **COMMENT** |
| --- | --- | --- |
| **Column1** | **Y/N** | **Column2** |
| **1. SPECIMEN** |  |  |
| Detailed description of specimen type and numbers | **Y** | Materials and methods |
| Sampling procedure (including time to storage) | **Y** | Materials and methods |
| Sample aliquotation, storage conditions and duration | **Y** | Materials and methods |
| **2. NUCLEIC ACID EXTRACTION** |  |  |
| Description of extraction method including amount of sample processed | **Y** | Materials and methods |
| Volume of solvent used to elute/resuspend extract | **Y** | Materials and methods |
| Number of extraction replicates | **Y** | Materials and methods |
| Extraction blanks included? | **Y** | Materials and methods |
| **3. NUCLEIC ACID ASSESSMENT AND STORAGE** |  |  |
| Method to evaluate quality of nucleic acids | **Y** | Materials and methods |
| Method to evaluate quantity of nucleic acids (including molecular weight and calculations when using mass) | **Y** | Materials and methods |
| Storage conditions: temperature, concentration, duration, buffer, aliquots | **Y** | Materials and methods |
| Clear description of dilution steps used to prepare working DNA solution | **Y** | Materials and methods |
| **4. NUCLEIC ACID MODIFICATION** | **N** | N/A |
| Template modification (digestion, sonication, pre-amplification, bisulphite etc.) | **N** | N/A |
| Details of repurification following modification if performed | **N** | N/A |
| **5. REVERSE TRANSCRIPTION** | **Y** | Materials and methods |
| cDNA priming method and concentration | **Y** | Materials and methods |
| One or two step protocol (include reaction details for two step) | **Y** | Materials and methods |
| Amount of RNA added per reaction | **Y** | Materials and methods |
| Detailed reaction components and conditions | **Y** | Materials and methods |
| Estimated copies measured with and without addition of RT* | **N** | None |
| Manufacturer of reagents used with catalogue and lot numbers | **Y** | Materials and methods |
| Storage of cDNA: temperature, concentration, duration, buffer and aliquots | **Y** | Materials and methods |
| **6. dPCR OLIGONUCLEOTIDES DESIGN AND TARGET INFORMATION** |  |  |
| Sequence accession number or official gene symbol | **Y** | S1 Table |
| Method (software) used for design and *in silico* verification | **Y** | Materials and methods |
| Location of amplicon | **N** | N/A |
| Amplicon length | **Y** | S1 Table |
| Primer and probe sequences (or amplicon context sequence)** | **Y** | S1 Table |
| Location and identity of any modifications | **N** | None |
| Manufacturer of oligonucleotides | **Y** | Materials and methods |
| **7. dPCR PROTOCOL** |  |  |
| Manufacturer of dPCR instrument and instrument model | **Y** | Materials and methods |
| Buffer/kit manufacturer with catalogue and lot number | **Y** | S2 Table |
| Primer and probe concentration | **Y** | Materials and methods |
| Pre-reaction volume and composition (incl. amount of template and if restriction enzyme added) | **N** | None |
| Template treatment (initial heating or chemical denaturation) | **N** | None |
| Polymerase identity and concentration, Mg++ and dNTP concentrations*** | **Y** | Materials and methods |
| Complete thermocycling parameters | **Y** | Materials and methods |
| **8. ASSAY VALIDATION** |  |  |
| Details of optimisation performed | **Y** | S8 Table |
| Analytical specificity (vs. related sequences) and limit of blank (LOB) | **N** | None |
| Analytical sensitivity/LoD and how this was evaluated | **Y** | S2 Fig |
| Testing for inhibitors (from biological matrix/extraction) | **N** | None |
| **9. DATA ANALYSIS** |  |  |
| Description of dPCR experimental design | **Y** | Materials and methods |
| Comprehensive details negative and positive of controls (whether applied for QC or for estimation of error) | **Y** | S2 Fig |
| Partition classification method (thresholding) | **Y** | Materials and methods |
| Examples of positive and negative experimental results (including fluorescence plots in supplemental material) | **Y** | S2 Fig |
| Description of technical replication | **Y** | Materials and methods |
| Repeatability (intra-experiment variation) | **Y** | S8 Table |
| Reproducibility (inter-experiment/user/lab etc. variation ) | **N** | N/A |
| Number of partitions measured (average and standard deviation ) | **Y** | S8 Table |
| Partition volume | **Y** | S2 Table |
| Copies per partition (λ or equivalent ) (average and standard deviation) | **Y** | S8 Table |
| dPCR analysis program (source, version) | **Y** | S2 Table |
| Description of normalisation method | **Y** | Materials and methods |
| Statistical methods used for analysis | **Y** | Materials and methods |
| Data transparency | raw data included as supplemental files: | S2 Fig and S8 Table |

Abbreviations: N/A, Not applicable.
