# Supplementary material for: Integrative proteomic and lipidomic analysis of GNB1 and SCARB2 knockdown in human subcutaneous adipocytes
Source: PLoS One. 2025 Mar 24;20(3):e0319163. doi: 10.1371/journal.pone.0319163 (PMC11932494; doi:10.1371/journal.pone.0319163)
Supplement: S8 Table — (DOCX) [file pone.0319163.s013.docx]

**S8 Table.** **Data analysis of digital PCR after gene knockdown.**

| **Gene** | **Sample name** | **Partitions (valid)^a^** | **Partitions (positive)^b^** | **Concentration (copies/μL)^c^** | **Mean**  **(copies/μL)** | **SD** |
| --- | --- | --- | --- | --- | --- | --- |
| *GNB1* | siRNA-nc_1 | 8,256 | 539 | 205.6 | 191.3 | 64.1 |
|  | siRNA-nc_2 | 8,243 | 314 | 121.2 |  |  |
|  | siRNA-nc_3 | 8,050 | 615 | 247.0 |  |  |
|  | siRNA-*GNB1*_1 | 8,261 | 78 | 29.6 | 27.4 | 3.5 |
|  | siRNA-*GNB1*_2 | 8,255 | 74 | 29.1 |  |  |
|  | siRNA-*GNB1*_3 | 8,260 | 60 | 23.4 |  |  |
|  | NTC^d^ | 8,253 | 0 | 0 | 0 | 0 |
| *SCARB2* | siRNA-nc_1 | 8,281 | 266 | 101.8 | 93.3 | 20.5 |
|  | siRNA-nc_2 | 8,261 | 175 | 70.0 |  |  |
|  | siRNA-nc_3 | 8,265 | 273 | 108.2 |  |  |
|  | siRNA-*SCARB2*_1 | 8,266 | 132 | 50.2 | 44.7 | 5.5 |
|  | siRNA-*SCARB2*_2 | 8,252 | 112 | 44.7 |  |  |
|  | siRNA-*SCARB2*_3 | 8,268 | 98 | 39.2 |  |  |
|  | NTC | 8,248 | 0 | 0 | 0 | 0 |
| *ACTB* | siRNA-nc_1 | 8,194 | 3,176 | 1,530.3 | 1,244.5 | 247.6 |
|  | siRNA-nc_2 | 8,208 | 2,386 | 1,110.1 |  |  |
|  | siRNA-nc_3 | 8,196 | 2,367 | 1,093.2 |  |  |
|  | siRNA-*GNB1*_1 | 8,229 | 1,723 | 732.9 | 690.7 | 41.1 |
|  | siRNA-*GNB1*_2 | 8,198 | 1,557 | 688.3 |  |  |
|  | siRNA-*GNB1*_3 | 8,223 | 1,478 | 650.8 |  |  |
|  | siRNA-*SCARB2*_1 | 8,258 | 4,153 | 2,219.5 | 1,898.9 | 306.4 |
|  | siRNA-*SCARB2*_2 | 8,188 | 3,115 | 1,609 |  |  |
|  | siRNA-*SCARB2*_3 | 8,172 | 3,537 | 1,868.3 |  |  |
|  | NTC | 8,252 | 0 | 0 | 0 | 0 |

^a^Total number of partitions in the digital PCR reaction.

^b^Number of positive partitions.

^c^The number of target gene copies (copies/μL) was calculated using Poisson statistics.

^d^NTC = no-template control.

Abbreviations: *GNB1,* G protein subunit beta 1; *SCARB2,* scavenger receptor class B member 2; *ACTB,* actin beta; siRNA-nc, siRNA-negative control; SD, standard deviation.
