# Supplementary material for: Integrative proteomic and lipidomic analysis of GNB1 and SCARB2 knockdown in human subcutaneous adipocytes
Source: PLoS One. 2025 Mar 24;20(3):e0319163. doi: 10.1371/journal.pone.0319163 (PMC11932494; doi:10.1371/journal.pone.0319163)
Supplement: S9 Table — (DOCX) [file pone.0319163.s014.docx]

**S9 Table.** **Data analysis of quantitative real-time PCR for adipogenic marker genes in *GNB1* and *SCARB2* knockdown cells.**

| **Gene** | **Sample name** | **Cq value (Replicate)** | | | **Cq**  **Mean** | **SD** | ***F*-value** | ***P*-value** | **FDR adjusted**  ***P*-value** |
| --- | --- | --- | --- | --- | --- | --- | --- | --- | --- |
|  |  | **1** | **2** | **3** |  |  |  |  |  |
| *PPARG* | siRNA-nc | 30.213680 | 30.632427 | 29.789942 | 30.212016 | 0.42 | 5.14 | 0.052 | 0.11 |
|  | siRNA-*GNB1* | 29.981297 | 30.418154 | 29.956799 | 30.118750 | 0.26 |  |  |  |
|  | siRNA-*SCARB2* | 29.093056 | 29.540184 | 29.371550 | 29.334930 | 0.23 |  |  |  |
|  | NTC | Undetermined | | | - | - |  |  |  |
| *CEBPA* | siRNA-nc | 30.532093 | 30.503506 | 31.238590 | 30.758063 | 0.42 | 0.23 | 0.790 | 0.79 |
|  | siRNA-*GNB1* | 31.093065 | 31.932583 | 31.977022 | 31.667557 | 0.50 |  |  |  |
|  | siRNA-*SCARB2* | 29.865759 | 30.151459 | 30.262823 | 30.093347 | 0.20 |  |  |  |
|  | NTC | 38.003563* | | | - | - |  |  |  |
| *FABP4* | siRNA-nc | 25.598566 | 25.698680 | 24.732225 | 25.343157 | 0.53 | 3.88 | 0.080 | 0.11 |
|  | siRNA-*GNB1* | 25.207409 | 25.239208 | 24.961897 | 25.136171 | 0.15 |  |  |  |
|  | siRNA-*SCARB2* | 23.956198 | 23.919004 | 24.150606 | 24.008603 | 0.12 |  |  |  |
|  | NTC | 37.551357* | | | - | - |  |  |  |
| *ADIPOQ* | siRNA-nc | 29.417887 | 29.326244 | 28.980892 | 29.241674 | 0.23 | 4.25 | 0.081 | 0.11 |
|  | siRNA-*GNB1* | 29.279823 | 29.258610 | 29.447230 | 29.328554 | 0.10 |  |  |  |
|  | siRNA-*SCARB2* | 28.497930 | 28.422146 | 28.770927 | 28.563668 | 0.18 |  |  |  |
|  | NTC | Undetermined | | | - | - |  |  |  |
| *ACTB* | siRNA-nc | 22.199148 | 22.705590 | 22.686468 | 22.530402 | 0.29 | - | - | - |
|  | siRNA-*GNB1* | 23.283289 | 23.258022 | 23.417265 | 23.319525 | 0.09 |  |  |  |
|  | siRNA-*SCARB2* | 21.749119 | 22.193426 | 21.930828 | 21.957791 | 0.22 |  |  |  |
|  | NTC | Undetermined | | | - | - |  |  |  |

Data were analyzed using the one-way ANOVA followed by false discovery rate (FDR) correction. Amplification was detected in the NTCs for *CEBPA* (Cq = 38.00) and *FABP4* (Cq = 37.55). According to MIQE guidelines, NTCs with Cq ≥ 40 can be ignored. While below 40, these values are substantially higher than those of the samples, indicating negligible contamination*. Abbreviations: *PPARG*, peroxisome proliferator activated receptor gamma; *CEBPA*, CCAAT enhancer binding protein alpha; *FABP4*, fatty acid binding protein 4; *ADIPOQ*, adiponectin, C1Q and collagen domain containing; *ACTB,* actin beta; NTC, no-template control; SD, standard deviation.
